# Higher bone cement volume in total knee arthroplasty lowers the risk of postoperative radiolucent lines

**DOI:** 10.1002/ksa.12582

**Published:** 2025-01-15

**Authors:** Maximilian Keintzel, Maria A. Smolle, Kevin Staats, Christoph Böhler, Reinhard Windhager, Amir Koutp, Andreas Leithner, Stefanie Donner, Carsten Perka, Tobias Reiner, Tobias Renkawitz, Alexandra Leica, Manuel Sava, Michael Hirschmann, Patrick Sadoghi

**Affiliations:** ^1^ Department of Orthopedics and Trauma‐Surgery Medical University of Vienna Vienna Austria; ^2^ Department of Orthopedics and Trauma Medical University of Graz Graz Austria; ^3^ Center for Musculoskeletal Surgery Charité – Universitätsmedizin Berlin Berlin Germany; ^4^ Department of Orthopaedics Heidelberg University Hospital Heidelberg Germany; ^5^ Department of Orthopaedic Surgery and Traumatology Kantonsspital Baselland Bruderholz Switzerland

**Keywords:** aseptic loosening, cementation technique, radiolucent lines, total knee arthroplasty

## Abstract

**Purpose:**

The aim of this multicenter study was to analyze the potential impact of patient demographics and cementation technique towards the development of radiolucent lines (RLLs) in primary total knee arthroplasty (TKA). It was hypothesized that cementation techniques, including higher cement volume, double‐layer cementation technique and hardening in full extension, reduce RLL incidence by improving stability, whereas demographic factors such as age, BMI and smoking may increase RLL risk by affecting bone quality and mechanical loading.

**Methods:**

Altogether, 776 patients (median age: 70.7 years; 39.2% males) underwent TKA at five tertiary orthopaedic centres between 11/2013 and 04/2023. X‐rays were analyzed retrospectively for the evaluation of RLLs taken between 6 and 36 months from surgery. RLLs on anterior–posterior and lateral X‐rays taken at a median of 14 months (range: 6–36) from primary surgery were evaluated using the Knee Society roentgenographic evaluation and scoring system. Potential associations of demographics and cementation technique on the occurrence of RLLs during follow‐up were analyzed with uni‐ and multivariate logistic regression models.

**Results:**

The overall incidence of RLLs around the TKA amounted to 37.4% (*n* = 290), with the tibial component (29.4%) being more commonly affected than the femoral component (15.0%). Patient age, gender, BMI and smoking habits were not significantly associated with higher incidence of RLLs (*p* > 0.05). The amount of cement used (odds ratio: 0.99; 95% confidence interval: 0.98–0.99; *p* = 0.028) was independently associated with a lower incidence of RLLs, irrespective of the double‐ versus single‐layer cementation technique, cement hardening in full extension and time required for the X‐ray.

**Conclusions:**

No influence of demographic data on the incidence of RLL was found, yet specific cementation techniques appeared beneficial. Future studies with longer follow‐up periods are required to provide further insight into the herein‐made preliminary findings and to assess potential associations with long‐term aseptic loosening rates.

**Level of Evidence:**

Level III, retrospective observational study.

**Abbreviations**
AE: Arbeitsgemeinschaft Endoprothetik
BMI: body mass index
CR: cruciate retaining
CT: computer tomography
OR: odds ratio
PS: posterior stabilized
RF: fixed bearing
RLL: radiolucent line
RP: rotating platform
SD: standard deviation
TKA: total knee arthroplasty

## INTRODUCTION

Aseptic loosening is one of the major reasons for implant revision in total knee arthroplasty (TKA) [22, 29, 30]. It is preceded by the occurrence of radiolucent lines (RLLs) at the implant/cement–bone interface on sequential anterior–posterior (AP) and lateral radiographs [7, 8, 20, 33, 37]. On initial postoperative X‐rays, RLLs are typically associated with inadequate cement penetration due to sclerotic bone quality, suboptimal bone cuts or misalignment and are usually located at the cement–bone interface [4, 37]. A progression of RLLs over time is often attributed to micromotion, followed by bone resorption and cement loosening [6, 11, 23]. Advances in pulsed lavage techniques and cement pressurization, as well as the use of tourniquets, have improved cement penetration into the bone [13, 18, 21, 28, 42]. However, recent evidence suggests that debonding takes place, particularly at the implant–cement interface [5, 18, 34].

Several factors are considered to affect the development of RLLs during follow‐up, including TKA design, bone density, demographic variables and surgical and cementation technique [4, 11, 14, 16, 24, 26, 31, 32, 34, 35]. The latter is of particular interest, given that double‐ versus single‐layer cement application, amount and type of cement used, single‐ versus two‐staged cementation, use of a tourniquet, meticulous irrigation of bone cuts and cement hardening in full extension versus minor flexion of the knee may all influence the implant–cement–bone attachment and, consecutively, the development of RLLs [3, 10, 12, 13, 27, 42].

The purpose of this multicenter study was to assess the potential influence of demographic data and cementation technique on development of RLLs in primary TKA. It was hypothesized that these factors influence the occurrence of RLLs, given that meticulous cementation techniques may reduce their occurrence by improving stability, while specific demographic factors potentially increase their occurrence by negatively affecting bone quality and mechanical loading.

## PATIENTS AND METHODS

**Figure 1:**
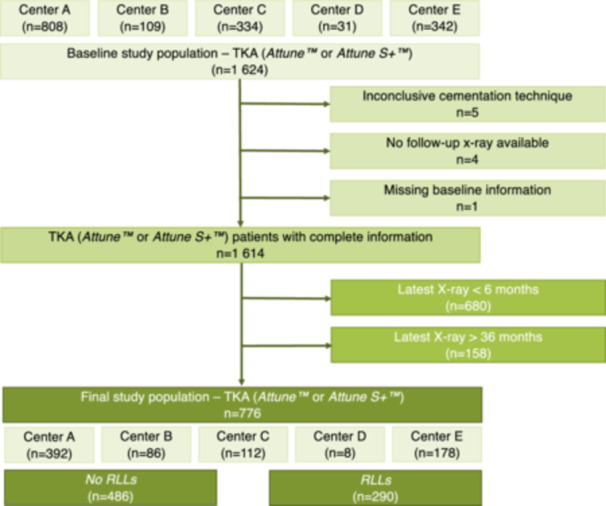
Flow chart showing the study selection process.

All patients receiving the same cemented TKA system (Attune™ Knee System; DePuy Synthes) between 11/2013 and 04/2023 at five tertiary orthopaedic centres were potentially eligible for this retrospective observational study. Patients must not have undergone previous knee surgeries, except for meniscectomy. During regular follow‐up visits as well as the latest follow‐up, AP and lateral radiographs of the treated knee joint were obtained in a standardized technique. Notably, only the most recent X‐ray was used for the evaluation, taken between a minimum of 6 and a maximum of 36 months following index surgery. Consequently, after the exclusion of five patients with inconclusive cementation technique, four lacking a sufficient follow‐up X‐ray, one with missing baseline information and 838 patients with X‐rays taken outside of the defined time frame (i.e., 6–36 months following surgery), 776 individuals were included (Figure 1). RLLs at the tibial component could not be assessed with certainty in one individual, resulting in 775 patients with information on tibial RLLs available. For cementation, either PALACOS® fast R + G (Heraeus; *n* = 768) or PALACOS® R (Heraeus; *n* = 8) had been used. The amount of cement used during each procedure was measured by the number of packages used, with each package containing 20 g of the respective cement. A tourniquet was applied during cementation in all patients. Bone cuts were thoroughly irrigated with high‐pressure jet lavage prior to drying and consecutive cementation of implant components using a cement gun and pre‐cooled vacuum‐mixed cement. Cement curing time was strictly awaited, and excess cement was removed, in adherence to the AE guidelines for cementation [40]. The double‐layer cementation technique was defined as coating both the undersurface of the tibial tray, including the keel, and the prepared bone prior to implantation.

All the latest X‐rays of the knee joint were assessed based on the Knee Society roentgenographic evaluation and scoring system by Ewald et al. [8, 32, 38]. Differences in the presence, extent and distribution of RLLs on the latest follow‐up X‐rays were assessed depending on patient demographics and the cementation technique employed. Notably, during the study period, a revised version of the tibial baseplate design (Attune S+™ tibial baseplate) was introduced into the market and was consecutively used at the participating centres. The influence of the tibial baseplate design on the occurrence of RLLs has already been published by this study group [31].

This study was approved by the local institutional review board (Institutional Review Board of the *Medical University of Graz, Austria*; IRB number: 30‐ 253 ex 17/18). Informed consent was not required as the data and images used were anonymized and nonidentifiable.

### Statistical analysis

Numbers are given with valid percentages. The Shapiro–Wilk test was used to assess the distribution of continuous variables. Means (with standard deviations, SD) and medians (with ranges) were provided for normally and nonnormally distributed continuous variables, respectively. Differences between groups were assessed using chi‐squared and Fisher's exact test. Variances in continuous parameters were analysed with *T*‐tests and Mann–Whitney‐*U*‐tests for normally and nonnormally distributed variables. Logistic regression analyses (univariate and multivariate) were applied to analyse differences between the presence of radiolucent lines and demographic factors as well as the cementation technique. All parameters with a *p* < 0.100 in the univariate analysis were included in the multivariate logistic regression model. Considering that the development of RLLs constitutes a time‐dependent event [9, 32], time required for the X‐ray was likewise included in the multivariate analysis. A two‐sided *p* < 0.05 was considered statistically significant.

## RESULTS

### Description of cohort

Overall, 304 patients were male (39.2%), and the median patient age at surgery was 70.7 (range: 23.8–100.0) years. Median amount of cement used was 40 g (range: 40–320 g). A double‐layer technique had been employed in 473 patients (61.0%) and staged cementation in 322 (41.6%). In the majority of TKAs (75.6%; 587/776), the knee was held in full extension upon cement hardening (Table 1). Further demographic and implant‐related data are visible in Table 1.

**Table 1:** Description of the study population, separated by the absence and presence of RLLs on the latest X‐ray.

	Entire cohort (*n* = 776)	No RLL (*n* = 486)	RLL (*n* = 290)	*p* value*
Demographics				
Male gender	304/776 (39.2%)	192/486 (39.5%)	112/290 (38.6%)	n.s.
Patient age (in years; median, range)	70.7 [23.8–100.0]	71.1 [23.8–91.5]	69.8 [76.6– 100.0]	n.s.
BMI (in years; median, range)	29.4 [15.8–53.4]	29.2 [15.8–48.4]	29.6 [17.7–53.4]	n.s.
Smoking	126/768 (16.4%)	84/481 (17.5%)	42/287 (14.6%)	n.s.
Time required for the X‐ray (in months; median, range)	14 [6–36]	14 [6–36]	15 [6–36]	n.s.
**Implant details & cementation technique**				
Implant configuration				n.s.
PS + FB	93/765 (12.2%)	61/478 (12.8%)	32/287 (11.2%)	
PS + RP	32/765 (4.2%)	23/478 (4.8%)	9/287 (3.1%)	
CR + FB	551/765 (72.0%)	338/478 (70.7%)	213/287 (74.2%)	
CR + RP	89/765 (11.6%)	56/478 (11.7%)	33/287 (11.5%)	
Double‐layer technique (vs. single‐layer technique)	473/776 (61.0%)	308/486 (63.4%)	165/290 (56.9%)	n.s.
Amount of cement used (in g, range)	40 [40–320]	40 [40–320]	40 [40– 280]	n.s.
Staged cementation – yes	322/775 (41.6%)	205/485 (42.3%)	117/290 (40.3%)	n.s.
Full extension (vs. 10° knee flexion)	587/776 (75.6%)	378/486 (77.8%)	209/290 (72.1%)	n.s.

*Note*: Numbers are given together with valid percentages.

Abbreviations: CR, cruciate retaining; FB, fixed bearing; n.s., not significant; PS, posterior stabilized; RLL, radiolucent line; RP, rotating platform.

* Chi‐squared test or Mann–Whitney‐*U*‐test, n.s. if *p* > 0.05.

### Radiolucent lines and associated factors

**Figure 2:**
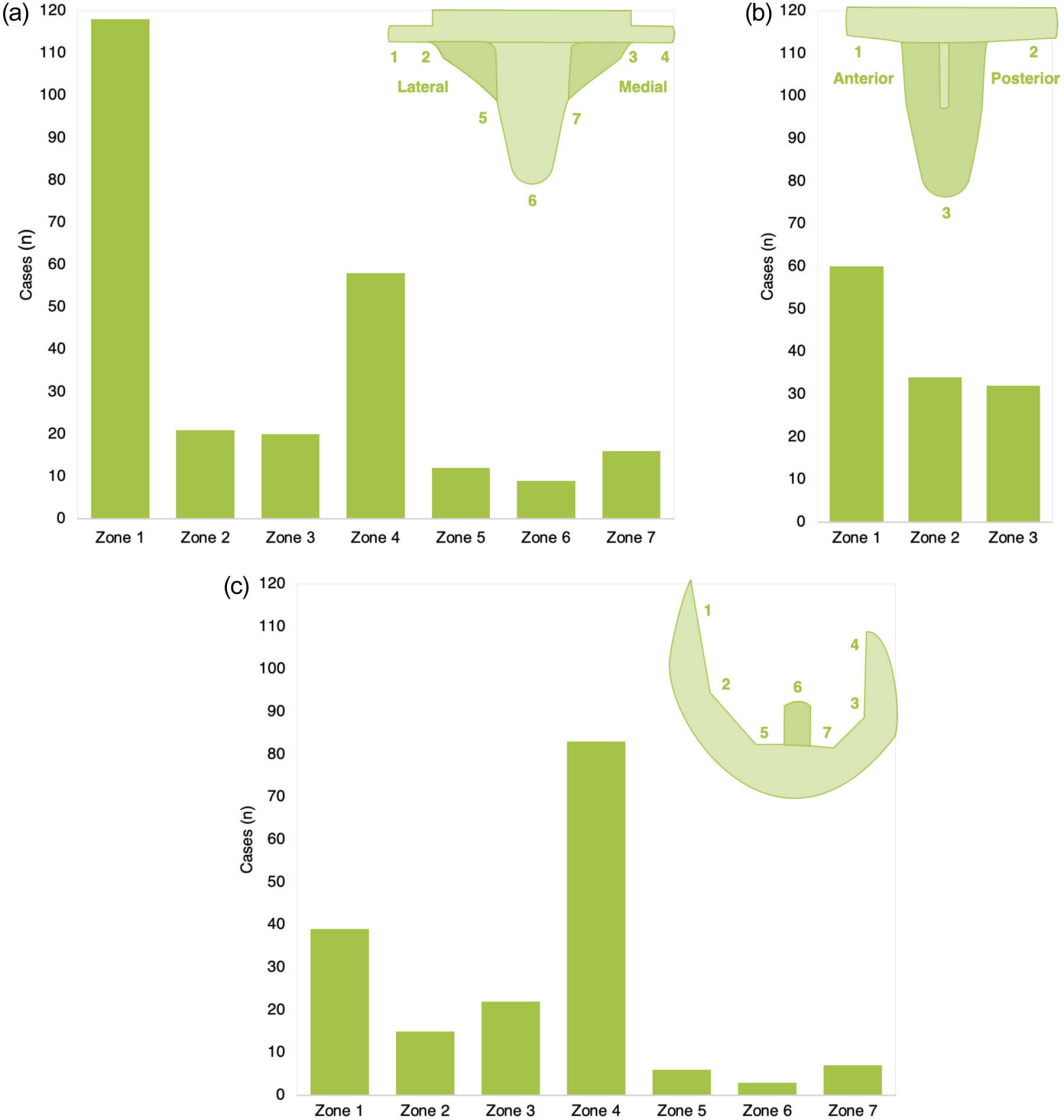
Frequency of radiolucent lines according to Ewald et al. Frequency of RLLs at the tibial component in the AP (a) and lateral (b) planes, as well as the lateral femoral plane (c).

RLLs around the TKA (including tibial and femoral components) were observed in 290 patients (37.4%) at a median follow‐up (i.e., time of X‐ray evaluation) of 14.0 months (range: 6–36). RLLs were more often seen at the tibial (29.4%) than the femoral component (15.0%; Figure 2).

The frequency of RLLs at different femoral and tibial zones is visible in Figure 2. No significant difference in patient demographics or follow‐up period depending on the absence or presence of RLLs on the latest X‐ray was found (Table 1). Notably, though, patients receiving the redesigned baseplate presented significantly fewer RLLs on the latest X‐ray compared to the previous design (*p* < 0.001; Table 1). Per tendency, a lower amount of cement had been used in patients later presenting with RLLs compared to those without (*p* = ns; Table 1), and rather a single‐layer technique had been employed (*p* = ns; Table 1).

In the univariate logistic regression model, the only factor significantly associated with the reduced likelihood of RLLs being present was a higher amount of cement (*p* = 0.005; Table 2). The double‐layer technique (*p* = ns) and cement hardening in full extension (*p* = ns; Table 2) were not significantly associated with a lower incidence of RLLs. In addition, none of the demographic or other implant‐ as well as cementation‐technique‐associated factors showed a significant association (Table 2).

**Table 2:** Univariate logistic regression analysis for any radiolucent line depends on demographics, implant details, cementation technique and time required for the X‐ray.

Any radiolucent line	OR	95% CI	*p* value*
Demographics			
Male gender	0.96	0.72–1.30	n.s.
Age at surgery (in years)	0.99	0.98–1.01	n.s.
BMI	0.99	0.97–1.03	n.s.
Smoking—yes	0.81	0.54–1.21	n.s.
Time required for the X‐ray (in months)	1.00	0.99–1.02	n.s.
**Implant details & cementation technique**			
Implant configuration (ref. PS + FB)			
PS + RP	0.75	0.31–1.80	n.s.
CR + FB	1.20	0.76–1.90	n.s.
CR + RP	1.12	0.61–2.06	n.s.
Double‐layer technique (vs. single‐layer technique)	0.76	0.57–1.03	n.s.
Amount of cement used (in g)	0.99	0.98–0.99	0.005
Staged cementation – yes	0.92	0.69–1.24	n.s.
Full extension (vs. 10° knee flexion)	0.74	0.53–1.03	n.s.

Abbreviations: BMI, body mass index; CI, confidence interval; CR, cruciate retaining; FB, fixed bearing; n.s., not significant; OR, odds ratio; PS, posterior stabilized; RP, rotating platform.

* n.s. if *p* > 0.05.

In the multivariate logistic regression model, which included all factors with a *p* < 0.1 in the univariate analysis, as well as time required for the X‐ray, a higher amount of cement used (odds ration [OR]: 0.99; 95% confidence interval [CI]: 0.98–0.99; *p* = 0.028) was independently associated with a lower likelihood of RLLs being present (Table 3). This association was irrespective of the double versus single‐layer cementation technique, cement hardening in full extension and time required for the X‐ray (Table 3).

**Table 3:** Multivariate logistic regression analysis for any radiolucent line depends on demographics, implant details, cementation technique and time required for the X‐ray.

Any radiolucent line	OR	95% CI	*p* value*
Double‐layer technique (vs. single‐layer technique)	0.84	0.62–1.14	n.s.
Amount of cement used (in g)	0.99	0.98–0.99	0.028
Full extension (vs. 10° knee flexion)	0.84	0.59–1.18	n.s.
Time required for the X‐ray (in months)	1.01	0.99–1.03	n.s.

Abbreviations: CI, confidence interval; n.s., not significant; OR, odds ratio.

* n.s. if *p* > 0.05.

## DISCUSSION

The most important finding of this study is that the amount of bone cement used during implantation was independently associated with a reduced incidence of RLLs, while demographic factors did not show a significant association.

According to this study, a higher amount of bone cement used during implantation was associated with a reduced risk for RLLs, independent of the double versus single‐layer technique, cement hardening in full knee extension and time required for the X‐ray. One may thus argue that a higher amount of cement enhances cement penetration into the bone and consecutively reduces the occurrence of RLLs [6, 23, 43]. In line with this, an experimental study discovered that a double‐layer cementation technique at the tibial component in combination with a higher amount of bone cement leads to a desired cement penetration depth within the tibial cancellous bone [25]. Two further experimental studies reported improved cement penetration when the double‐layer cementation technique had been employed [36, 39]. However, we were unable to demonstrate a significant association between the single‐ and double‐layer cementation technique and an altered occurrence of RLLs, a per tendency lower incidence was found in case the double‐layer cementation technique had been used.

On the other hand, no significant association between demographic factors (gender, BMI, age or smoking habit) and the occurrence of RLLs on the latest follow‐up X‐rays was found. This is of particular interest, given that younger, physically active patients, and in this context especially males, may be at a higher risk for aseptic TKA loosening due to long‐term implant overload [9]. In contrast to the present results, Hoskins et al. reported that the probability of RLLs increases by 8% per BMI unit [15], and Abdel et al. demonstrated that the risk of aseptic failure of the tibial component is two‐fold higher in patients with a BMI of ≥35 kg/m^2^, regardless of age or coronal alignment [1]. In addition, while it is generally accepted that complication rates are higher in smokers compared to nonsmokers [2, 19], the influence of smoking on aseptic loosening or development of RLL following TKA remains unclear in literature [17]. In accordance, smoking status was not significantly associated with altered incidence of RLLs in the present study.

Some limitations have to be considered when interpreting the findings of the current study. First, the analyses are based on a retrospective cohort of TKA patients receiving the same implant design at five orthopaedic units. Although all participating centres have a high experience in TKA in general, as well as the herein analyzed implant in particular, it cannot be ruled out that small differences in local policies regarding cement preparation and surgical techniques have biased results obtained. In line with this, one has to consider that different surgeons at the centres were involved in the treatment of patients and that the centres contributed to the study with differing numbers of cases (Figure 1). Second, only the latest X‐ray during follow‐up, truncated at 36 months, was used for the evaluation of RLLs. Therefore, information on potential dynamic changes of RLLs, as well as on the occurrence of RLLs beyond 36 months, cannot be provided. In addition, differing follow‐up periods ranging from 6 to 36 months from index TKA may have influenced the incidence of RLLs, given that their development is considered time‐dependent [9, 32]. To account for this potential bias, time required for the latest X‐ray (equivalent to the latest follow‐up) was included in the multivariate analyses. Moreover, in this study, no differentiation between RLLs at the cement–implant and cement–bone interfaces was made, given that radiographs had been used. While they reliably allow the detection of RLLs, differentiation of fine details is impaired [41, 44]. Thus, future studies combining both immediate postoperative imaging and advanced imaging techniques as CT scans may further clarify the dynamic nature and location‐specific progression of RLLs.

## CONCLUSIONS

A higher amount of bone cement used during TKA implantation was associated with a lower risk of RLLs occurring during follow‐up. On the other hand, no influence of demographic factors was found. Future studies with longer follow‐up periods are required to shed additional light on the preliminary findings herein made and to detect potential associations with aseptic loosening risk in the long term.

## AUTHOR CONTRIBUTIONS

Maximilian Keintzel and Maria A. Smolle wrote the main manuscript. Maria A. Smolle performed statistical analyses and compiled tables as well as figures. Patrick Sadoghi was responsible for study conceptualization. Kevin Staats, Christoph Böhler, Alexandra Leica and Patrick Sadoghi supervised the work. Data collection was carried out by Amir Koutp, Maximilian Keintzel, Stefanie Donner, Tobias Reiner, Alexandra Leica and Manuel Sava. All authors (Maximilian Keintzel, Maria A. Smolle, Kevin Staats, Christoph Böhler, Reinhard Windhager, Amir Koutp, Andreas Leithner, Stefanie Donner, Carsten Perka, Tobias Reiner, Tobias Renkawitz, Alexandra Leica, Manuel Sava, Michael Hirschmann and Patrick Sadoghi) reviewed and revised the main manuscript.

## CONFLICT OF INTEREST STATEMENT

M.A.S. has received travel grants from Alphamed Fischer, Implantcast, ImplanTec and PharmaMar, outside of the submitted work. C.B. has received payments/honoraria from Medacta and DePuy Synthes, outside of the submitted work, and declares leadership roles in the Executive Board Austrian Society of Orthopaedics, the Executive Board ComGen of the German Arthroplasty Society and Arthroplasty Society of the Austrian Society for Orthopaedics and Traumatology. R.W. has received consulting fees from Johnson&Johnson, Medacta, Stryker European and Operations Limited, outside of the submitted work, and reports other financial/non‐financial interests by DePuy Synthes (product development) and Johnson&Johson (life case observation, educational agreement). A.L. has received institutional educational grants from Alphamed, Medacta and Johnson&Johnson, outside of the submitted work. S.D. has received payments/honoraria from Medacta and DePuy Synthes, has received payment for expert testimony as well as travel support from Smith & Nephew—Female Global Advisory Group, outside of the submitted work, and declares leadership role in Smith & Nephew—Female Global Advisory Group, Enovis Mathys and Johnson/Johnson DePuy. C.P. declares royalties/licenses from DePuy Synthes, Smith & Nephew and Zimmer, has received consulting fees from DePuy Synthes and Zimmer, declares payments/honoraria from Zimmer, DePuy Synthes and Ethicon (made to institution), outside of the submitted work, and has leadership roles in the Bone&Joint Journal, International Hip Society and DGOOC. T.B. has received payments/honoraria from Arbeitsgemeinschaft Endoprothetik (AE), Aesculap, DGOU, Zimmer, DGOOC, Stiftung Oskar Helene Heim Berlin, BVOU, Vielberth Foundation Regensburg, DePuy International, The German Ministry of Education and Research, Otto Bock Foundation, German Federal Ministry of Economic Cooperation and Development, Deutsche Arthrose Hilfe and Deutsche Forschungsgemeinschaft, outside of the submitted work, and reports board membership of the German Society for Orthopaedics and Trauma (DGOOC) as well as Vice presidency of the Professional Association of Orthopaedic Specialists and Trauma Surgeons (BVOU). M.T.H. has received consulting fees from DePuy Synthes and Symbios, payments/honoraria from DePuy Synthes, Symbios and Smith & Nephew, support for attending meetings/travel from DePuy Synthes, Smith & Nephew and Symbios, outside of the submitted work, has participated on a Data Safety Monitoring Board/Advisory Board for DePuy Synthes and declares leadership or fiduciary roles in the KSSTA journal, ESSKA, German Knee Society and Personalized Arthroplasty Society. P.S. has received consulting fees and payments/honoraria from DePuy Synthes and Medacta, support for attending meetings/travel from DePuy Synthes, Medacta and Alphamed, outside of the submitted work, has participated on a Data Safety Monitoring Board/Advisory Board for DePuy Synthes and declares leadership/fiduciary role in the KSSTA journal. The remaining coauthors (M.K., K.S., A.K., T.B., A.L., M.S.) have no conflicts of interest to declare.

## ETHICS STATEMENT

This study was approved by the local institutional review board (Institutional Review Board of the *Medical University of Graz, Austria*; IRB number: 30‐253 ex 17/18). Patient informed consent was not required as the data and images used were anonymized and non‐identifiable.

## Data Availability

The underlying data of this study are available upon reasonable request from the corresponding author.

## References

[1] Abdel MP , Bonadurer GF , Jennings MT , Hanssen AD . Increased aseptic tibial failures in patients with a BMI ≥35 and well‐aligned total knee arthroplasties. J Arthroplasty. 2015;30:2181–2184. 26220103 10.1016/j.arth.2015.06.057

[2] Bedard NA , Dowdle SB , Wilkinson BG , Duchman KR , Gao Y , Callaghan JJ . What is the impact of smoking on revision total knee arthroplasty? J Arthroplasty. 2018;33:S172–S176. 29680584 10.1016/j.arth.2018.03.024

[3] Berend ME , Ritter MA , Meding JB , Faris PM , Keating EM , Redelman R , et al. The Chetranjan Ranawat Award: tibial component failure mechanisms in total knee arthroplasty. Clin Orthop Relat Res. 2004;428:26–34. 10.1097/01.blo.0000148578.22729.0e 15534515

[4] Billi F , Kavanaugh A , Schmalzried H , Schmalzried TP . Techniques for improving the initial strength of the tibial tray‐cement interface bond. Bone Joint J. 2019;101–b:53–58. 10.1302/0301-620X.101B1.BJJ-2018-0500.R1 30648489

[5] Bonutti P , Khlopas A , Chughtai M , Cole C , Gwam C , Harwin S , et al. Unusually high rate of early failure of tibial component in ATTUNE total knee arthroplasty system at implant–cement interface. J Knee Surg. 2017;30:435–439. 28591930 10.1055/s-0037-1603756

[6] Cawley DT , Kelly N , McGarry JP , Shannon FJ . Cementing techniques for the tibial component in primary total knee replacement. Bone Joint J. 2013;95–b:295–300. 10.1302/0301-620X.95B3.29586 23450010

[7] Elmallah RK , Scuderi GR , Jauregui JJ , Meneghini RM , Dennis DA , Backstein DB , et al. Radiographic evaluations of revision total knee arthroplasty: a plea for uniform assessments. J Arthroplasty. 2015;30:1981–1984. 26364904 10.1016/j.arth.2015.08.013

[8] Ewald FC . The Knee Society total knee arthroplasty roentgenographic evaluation and scoring system. Clin Orthop Relat Res. 1989;248:9–12. 2805502

[9] Gallo J , Goodman SB , Konttinen YT , Wimmer MA , Holinka M . Osteolysis around total knee arthroplasty: a review of pathogenetic mechanisms. Acta Biomater. 2013;9:8046–8058. 23669623 10.1016/j.actbio.2013.05.005 PMC4003873

[10] Gapinski ZA , Yee EJ , Kraus KR , Deckard ER , Meneghini RM . The effect of tourniquet use and sterile carbon dioxide gas bone preparation on cement penetration in primary total knee arthroplasty. J Arthroplasty. 2019;34:1634–1639. 31010776 10.1016/j.arth.2019.03.050

[11] Guha AR , Debnath UK , Graham NM . Radiolucent lines below the tibial component of a total knee replacement (TKR)—a comparison between single‐and two‐stage cementation techniques. Int Orthop. 2008;32:453–457. 17364179 10.1007/s00264-007-0345-6 PMC2532273

[12] Han J , Zhang XY , Mu SY , Liu SL , Cui QT , Zhang C , et al. Tourniquet application in primary total knee arthroplasty for osteoarthritis: a systematic review and meta‐analysis of randomized controlled trials. Front Surg. 2022;9:994795. 36684363 10.3389/fsurg.2022.994795 PMC9852050

[13] Hegde V , Bracey DN , Johnson RM , Dennis DA , Jennings JM . Tourniquet use improves cement penetration and reduces radiolucent line progression at 5 years after total knee arthroplasty. J Arthroplasty. 2021;36:S209–S214. 33500203 10.1016/j.arth.2020.12.048

[14] Holzer LA , Finsterwald MA , Sobhi S , Jones CW , Yates PJ . Application of bone cement directly to the implant in primary total knee arthroplasty. Short‐term radiological and clinical follow‐up of two different cementing techniques. Arch Orthop Trauma Surg. 2023;144:333–340. 37736767 10.1007/s00402-023-05057-9

[15] Hoskins W , Gorup P , Claireaux H , Stokes C , Bingham R . High incidence of radiolucent lines at the implant–cement interface of a new total knee replacement. ANZ J Surg. 2020;90:1299–1302. 32536016 10.1111/ans.16046

[16] Jaeger S , Eissler M , Schwarze M , Schonhoff M , Kretzer JP , Bitsch RG . Early tibial loosening of the cemented ATTUNE knee arthroplasty—just a question of design? Knee. 2021;30:170–175. 33933907 10.1016/j.knee.2021.01.003

[17] Kapadia BH , Johnson AJ , Naziri Q , Mont MA , Delanois RE , Bonutti PM . Increased revision rates after total knee arthroplasty in patients who smoke. J Arthroplasty. 2012;27:1690–1695. 22633104 10.1016/j.arth.2012.03.057

[18] Martin JR , Otero JE , Mason JB , Fehring TK . Where is the “Weak Link” of fixation in contemporary cemented total knee replacements? J Arthroplasty. 2021;36:2497–2501. 33676813 10.1016/j.arth.2021.02.029

[19] Matharu GS , Mouchti S , Twigg S , Delmestri A , Murray DW , Judge A , et al. The effect of smoking on outcomes following primary total hip and knee arthroplasty: a population‐based cohort study of 117,024 patients. Acta Orthop. 2019;90:559–567. 31370730 10.1080/17453674.2019.1649510 PMC6844375

[20] Meneghini RM , Mont MA , Backstein DB , Bourne RB , Dennis DA , Scuderi GR . Development of a modern knee society radiographic evaluation system and methodology for total knee arthroplasty. J Arthroplasty. 2015;30:2311–2314. 26122112 10.1016/j.arth.2015.05.049

[21] O'Donovan P , McAleese T , Harty J . Does lucency equate to revision? A five‐year retrospective review of Attune and Triathlon total knee arthroplasty. Knee Surg Sports Traumatol Arthrosc. 2023;31:4773–4781. 37516985 10.1007/s00167-023-07509-6 PMC10598109

[22] Piedade SR , Pinaroli A , Servien E , Neyret P . Revision after early aseptic failures in primary total knee arthroplasty. Knee Surg Sports Traumatol Arthrosc. 2009;17:248–253. 19082578 10.1007/s00167-008-0667-y

[23] Refsum AM , Nguyen UV , Gjertsen J‐E , Espehaug B , Fenstad AM , Lein RK , et al. Cementing technique for primary knee arthroplasty: a scoping review. Acta Orthop. 2019;90:582–589. 31452416 10.1080/17453674.2019.1657333 PMC6844414

[24] Ritter MA , Herbst SA , Keating EM , Faris PM . Radiolucency at the bone‐cement interface in total knee replacement. The effects of bone‐surface preparation and cement technique. J Bone Joint Surg. 1994;76:60–65. 8288666 10.2106/00004623-199401000-00008

[25] Rodríguez‐Collell JR , Mifsut D , Ruiz‐Sauri A , Rodríguez‐Pino L , González‐Soler EM , Valverde‐Navarro AA . Improving the cementation of the tibial component in knee arthroplasty: a study of four techniques in the cadaver. Bone Joint Res. 2021;10:467–473. 34340533 10.1302/2046-3758.108.BJR-2020-0524.R1 PMC8414436

[26] Sadoghi P , Leithner A , Weber P , Friesenbichler J , Gruber G , Kastner N , et al. Radiolucent lines in low‐contact‐stress mobile‐bearing total knee arthroplasty: a blinded and matched case control study. BMC Musculoskelet Disord. 2011;12:142. 21714916 10.1186/1471-2474-12-142 PMC3152942

[27] Sasaki R , Nagashima M , Otani T , Okada Y , Aibara N , Takeshima K , et al. Pressurized carbon dioxide lavage reduces the incidence of a radiolucent line around the tibial component two years after total knee arthroplasty. J Orthop Surg. 2022;17:349. 10.1186/s13018-022-03204-3 PMC9284780 35841041

[28] Schlegel UJ , Siewe J , Delank KS , Eysel P , Püschel K , Morlock MM , et al. Pulsed lavage improves fixation strength of cemented tibial components. Int Orthop. 2011;35:1165–1169. 20953784 10.1007/s00264-010-1137-y PMC3167426

[29] Sharkey PF , Lichstein PM , Shen C , Tokarski AT , Parvizi J . Why are total knee arthroplasties failing today‐‐has anything changed after 10 years? J Arthroplasty. 2014;29:1774–1778. 25007726 10.1016/j.arth.2013.07.024

[30] Skwara A , Figiel J , Knott T , Paletta JRJ , Fuchs‐Winkelmann S , Tibesku CO . Primary stability of tibial components in TKA: in vitro comparison of two cementing techniques. Knee Surg Sports Traumatol Arthrosc. 2009;17:1199–1205. 19572121 10.1007/s00167-009-0849-2

[31] Smolle MA , Keintzel M , Staats K , Böhler C , Windhager R , Koutp A , et al. Radiolucent lines and revision risk in total knee arthroplasty using the conventional versus the Attune S+ tibial baseplate. Results of a multicentre observational study. Bone Joint J. 2024;106:1–9. 10.1302/0301-620X.106B11.BJJ-2024-0084.R3 39481434

[32] Staats K , Wannmacher T , Weihs V , Koller U , Kubista B , Windhager R . Modern cemented total knee arthroplasty design shows a higher incidence of radiolucent lines compared to its predecessor. Knee Surg Sports Traumatol Arthrosc. 2019;27:1148–1155. 30244340 10.1007/s00167-018-5130-0 PMC6435629

[33] Stevens DG , Beharry R , McKee MD , Waddell JP , Schemitsch EH . The long‐term functional outcome of operatively treated tibial plateau fractures. J Orthop Trauma. 2001;15:312–320. 11433134 10.1097/00005131-200106000-00002

[34] Torino D , Damsgaard C , Kolessar DJ , Hayes DS , Foster B , Constantino J , et al. Tibial baseplate‐cement interface debonding in the ATTUNE total knee arthroplasty system. Arthroplast Today. 2022;17:165–171. 36164312 10.1016/j.artd.2022.06.012 PMC9508148

[35] Vaninbroukx M , Labey L , Innocenti B , Bellemans J . Cementing the femoral component in total knee arthroplasty: which technique is the best? Knee. 2009;16:265–268. 19138857 10.1016/j.knee.2008.11.015

[36] Vanlommel J , Luyckx JP , Labey L , Innocenti B , De Corte R , Bellemans J . Cementing the tibial component in total knee arthroplasty. J Arthroplasty. 2011;26:492–496. 20381290 10.1016/j.arth.2010.01.107

[37] Wautier D , Ftaïta S , Thienpont E . Radiolucent lines around knee arthroplasty components: a narrative review. Acta Orthop Belg. 2020;86:82–94. 32490778

[38] Wautier D , Thienpont E . Tibial implant design in primary TKA: retrospective comparison of two designs for the occurrence of radiolucent lines and aseptic loosening. Arch Orthop Trauma Surg. 2023;144:323–332. 37733127 10.1007/s00402-023-05030-6

[39] Wetzels T , van Erp J , Brouwer RW , Bulstra SK , van Raay JJAM . Comparing cementing techniques in total knee arthroplasty: an in vitro study. J Knee Surg. 2019;32:886–890. 30189440 10.1055/s-0038-1669917

[40] Wirtz D . AE‐Manual der Endoprothetik: Knie. Heidelberg: Springer Berlin. 10.1007/978-3-642-12889-92011

[41] Woisetschläger M , Booij R , Tesselaar E , Oei EHG , Schilcher J . Improved visualization of the bone‐implant interface and osseointegration in ex vivo acetabular cup implants using photon‐counting detector CT. Eur Radiol Exp. 2023;7:19. 37121937 10.1186/s41747-023-00335-y PMC10149426

[42] Xu H , Chen AF , Shoji MM , Fitz W , Lange JK . Are there more radiolucent lines in patients who underwent total knee arthroplasty with or without a tourniquet during cementation at 5 to 8 years after surgery? J Arthroplasty. 2023;38:1052–1056. 36858126 10.1016/j.arth.2023.02.057

[43] Cox ZC , Engstrom SM , Shinar AA , Polkowski GG , Mason JB , Martin JR . Is cement mantle thickness a primary cause of aseptic tibial loosening following primary total knee arthroplasty? Knee. 2023;40:305–312. 36592499 10.1016/j.knee.2022.12.003

[44] Zotti MGT , Campbell DG , Woodman R . Detection of periprosthetic osteolysis around total knee arthroplasties. J Arthroplasty. 2012;27:317–322. 21641179 10.1016/j.arth.2011.03.047

